# Multi-parameter approach to evaluate the timing of memory status after 17DD-YF primary vaccination

**DOI:** 10.1371/journal.pntd.0006462

**Published:** 2018-06-07

**Authors:** Christiane Costa-Pereira, Ana Carolina Campi-Azevedo, Jordana Grazziela Coelho-dos-Reis, Vanessa Peruhype-Magalhães, Márcio Sobreira Silva Araújo, Lis Ribeiro do Vale Antonelli, Cristina Toscano Fonseca, Jandira Aparecida Lemos, Luiz Cosme Cote Malaquias, Matheus de Souza Gomes, Laurence Rodrigues Amaral, Maria Rios, Caren Chancey, Harold Richard Persi, Jorge Marcelo Pereira, Maria de Lourdes de Sousa Maia, Marcos da Silva Freire, Reinaldo de Menezes Martins, Akira Homma, Marisol Simões, Anna Yoshida Yamamura, Roberto Henrique Guedes Farias, Alessandro Pecego Martins Romano, Carla Magda Domingues, Pedro Luiz Tauil, Pedro Fernando Costa Vasconcelos, Iramaya Rodrigues Caldas, Luiz Antônio Camacho, Andrea Teixeira-Carvalho, Olindo Assis Martins-Filho

**Affiliations:** 1 Centro de Pesquisas René Rachou, FIOCRUZ, Belo Horizonte, Minas Gerais, Brazil; 2 Secretaria de Estado de Saúde, Governo do Estado de Minas Gerais, Belo Horizonte, Minas Gerais, Brasil; 3 Universidade Federal de Alfenas, Alfenas, Minas Gerais, Brazil; 4 Laboratório de Bioinformática e Análises Moleculares, Universidade Federal de Uberlândia, Uberlândia, Minas Gerais, Brazil; 5 Center for Biologics Evaluation and Research – CBER – Food and Drug Administration (FDA), Silver Spring, Maryland, United States of America; 6 Instituto de Biologia do Exército, Rio de Janeiro, Rio de Janeiro, Brazil; 7 Instituto de Tecnologia em Imunobiológicos Bio-Manguinhos- FIOCRUZ, Rio de Janeiro, Rio de Janeiro, Brazil; 8 Secretaria de Vigilância em Saúde, Ministério da Saúde, Brasília, Federal District, Brazil; 9 Universidade de Brasília, Brasília, Federal District, Brazil; 10 Instituto Evandro Chagas– IEC, Ananindeua, Pará, Brazil; 11 Diretoria Regional de Brasília – DIREB, FIOCRUZ, Brasília, Federal District, Brazil; 12 Escola Nacional de Saúde Pública, FIOCRUZ, Rio de Janeiro, Rio de Janeiro, Brazil; University of California, Berkeley, UNITED STATES

## Abstract

In this investigation, machine-enhanced techniques were applied to bring about scientific insights to identify a minimum set of phenotypic/functional memory-related biomarkers for post-vaccination follow-up upon yellow fever (YF) vaccination. For this purpose, memory status of circulating T-cells (Naïve/early-effector/Central-Memory/Effector-Memory) and B-cells (Naïve/non-Classical-Memory/Classical-Memory) along with the cytokine profile (IFN/TNF/IL-5/IL-10) were monitored before-NV(day0) and at distinct time-points after 17DD-YF primary vaccination—PV(day30-45); PV(year1-9) and PV(year10-11). A set of biomarkers (eEfCD4; EMCD4; CMCD19; EMCD8; IFNCD4; IL-5CD8; TNFCD4; IFNCD8; TNFCD8; IL-5CD19; IL-5CD4) were observed in PV(day30-45), but not in NV(day0), with most of them still observed in PV(year1-9). Deficiencies of phenotypic/functional biomarkers were observed in NV(day0), while total lack of memory-related attributes was observed in PV(year10-11), regardless of the age at primary vaccination. Venn-diagram analysis pre-selected 10 attributes (eEfCD4, EMCD4, CMCD19, EMCD8, IFNCD4, IL-5CD8, TNFCD4, IFNCD8, TNFCD8 and IL-5CD4), of which the overall mean presented moderate accuracy to discriminate PV(day30-45)&PV(year1-9) from NV(day0)&PV(year10-11). Multi-parameter approaches and decision-tree algorithms defined the EMCD8 and IL-5CD4 attributes as the top-two predictors with moderated performance. Together with the PRNT titers, the top-two biomarkers led to a resultant memory status observed in 80% and 51% of volunteers in PV(day30-45) and PV(year1-9), contrasting with 0% and 29% found in NV(day0) and PV(year10-11), respectively. The deficiency of memory-related attributes observed at PV(year10-11) underscores the conspicuous time-dependent decrease of resultant memory following17DD-YF primary vaccination that could be useful to monitor potential correlates of protection in areas under risk of YF transmission.

## Author summary

In this study, a set of immunological biomarkers was studied in order to understand protection upon vaccination with yellow fever (17DD-YF) vaccine. For this purpose, the immunological memory statuses of circulating T- and B-cells along with the plasmatic molecules (cytokine profile) were monitored before and at distinct time-points after primary vaccination. A set of biomarkers were measured in the peripheral blood of primary 17-DD vaccinees after 30–45 days of vaccination, which were relatively sustained in vaccinees after 1–9 years of primary vaccination. Deficiencies and a total lack of memory-related immunological responses to yellow fever virus were observed after 10 to 11 years post-vaccination, regardless of the age at primary vaccination. Multi-parameter approaches defined two biomarkers (EMCD8 and IL-5CD4) as the top-two predictors of protection. The deficiency of attributes observed after 10–11 years post-vaccination reveals a time-dependent decrease of immunological memory responses related to the 17DD-YF vaccination. Therefore, these results highly suggest the need for close attention to vaccinees in YF endemic areas with more than 10 years of vaccination. At last, the biomarkers proposed in this study could be useful to monitor protection in YF-vaccinees living in or travelling to areas under risk of YF transmission.

## Introduction

The yellow fever (YF) vaccine is a live attenuated virus isolated in the 1930s, which has been considered the most robust and successful vaccine ever developed [1].

The YF primary vaccination triggers multilineage and polyfunctional immune responses, mediated by long-lasting neutralizing antibodies and a robust cellular immunity, comprised of mixed pro-inflammatory/regulatory profile [2–6]. Indeed, YF primary vaccination elicits an integrated immune response, including several arms of innate and adaptive immunity such as early CD4^+^T-cell response followed by a vigorous B-cell activation and long-term memory CD8^+^ T-cells [7–9].

The presence of neutralizing antibodies has been considered the reference for assessment of protection upon YF vaccination [10]. Although YF-specific neutralizing antibodies remain detectable by plaque reducing neutralization test (PRNT) for several years after a single-dose vaccination [4,11–13], it has been reported that the PRNT may reach critical levels after many years of primary YF vaccination, with seropositivity dropping to approximately 75.0%-78.4% in single dose vaccines [4,12,13].

Moreover, the quality of cellular memory seems to display an over-time decrease. Decreased levels of TNF-α and IFN-γ produced by CD4^+^ and CD8^+^ T-cells and increased levels of IL-10^+^ CD4^+^ T-cells were found in primary 17DD-YF vaccinees over time [6]. These findings focusing on the long-term persistence of effective memory T and B-cells, especially beyond 5–10 years after primary YF-17DD vaccination have provided invaluable insights into memory T-cell loss after 17DD-YF primary vaccination [6]. The translation of these findings towards practical laboratory tools would be relevant to identify potential correlates of protection to monitor the 17DD-YF vaccinees overtime.

In the present study, polychromatic flow cytometry was used to characterize phenotypic/functional biomarkers to define immune response signature and resultant memory status in a cohort of 139 volunteers, in a timeline from 30–45 days to 10–11 years after 17DD-YF primary vaccination. To determine the most suitable biomarkers to evaluate the long-term resultant memory upon YF primary vaccination, we first selected the potential correlates of protection at 30–45 days and filtered those with a persistent pattern within 1–9 years after primary vaccination. Machine-enhanced techniques were applied to bring about scientific insights to identify the minimum set of phenotypic/functional biomarkers for post-vaccination follow-up upon YF primary vaccination.

Our findings demonstrated that EMCD8 and IL-5CD4 are the top-two biomarkers that together with the PRNT titers can provide potential correlates of protection as the resultant memory following 17DD-YF primary vaccination. The decrease of these biomarkers overtime demonstrated a conspicuous time-dependent reduction of anti-17DD-YF resultant memory.

## Materials and methods

### Ethics statement

This study was performed by the Collaborative Group for Studies of Yellow Fever Vaccine upon approval by the Ethics Committee for studies with human subjects at Centro de Pesquisas René Rachou—CPqRR/FIOCRUZ (Protocol#24/2010). All subjects enrolled in the present investigation have signed the written consent form and were informed about the study before providing their written consent and blood collection.

### Study population

The study population consisted of non-vaccinated subjects (n = 46), males with age ranging from 18–19 years, referred as NV(day0) and three groups of primary vaccinated volunteers, categorized according to the time (days or years) after 17DD-YF vaccination, referred as: PV(day30-45); (n = 46, males, with age ranging from 18–19 years), PV(year1-9); (n = 48, 23 males and 25 females, age ranging from 20–59 years) and PV(year10-11);(n = 45, 38 males and 7 females, age ranging from 31–74 years).

The experimental design was structured as a paired longitudinal arm to identify early potential correlates of protection that included the groups NV(day0) and PV(day30-45), comprising army military recruits (RJ, Brazil). In addition, a cross-sectional arm, which included PV(year1-9) and PV(year10-11), comprised healthy volunteers resident of Alfenas (MG, Brazil).

Volunteers with seropositive results at baseline (PRNT>2.9 log_10_mIU/mL) were excluded from the study. Immunization cards confirmed primary vaccination. All subjects enrolled in the present investigation have signed the consent form. Five mL of peripheral whole blood were collected from each volunteer without anticoagulant for plaque-reduction neutralization test (PRNT) and twenty mL were collected in heparin for phenotypic and functional analyses.

### Plaque reduction neutralization test (PRNT)

17DD-YF neutralizing antibodies were measured by micro-PRNT Test as described previously by Simões and colleagues [14]. The tests were performed at Laboratório de Tecnologia Virológica, Bio-Manguinhos (LATEV, FIOCRUZ-RJ, Brazil). Titers of anti-YF neutralizing antibodies were expressed in Log mIU/mL. Samples were considered seropositive when PRNT titers were higher than 2.9 Log mIU/mL.

### Long-term 17DD-YF antigen recall of peripheral blood mononuclear cells *in vitro*

Peripheral blood mononuclear cells (PBMC) were obtained from heparinized whole blood samples by density Histopaque-gradient (Sigma, St Louis, MO, USA) centrifugation, as described by the manufacturer. Replicates of PBMC cultures (1.0x10^6^/well) were incubated in the presence of RPMI-1640 (Gibco, Grand Island, NY, USA), supplemented with 5% heat-inactivated AB normal human serum, 10,000IU/mL penicillin, 10mg/mL streptomycin and 0.025mg/mL amphotericin B (Sigma, St Louis, MO, USA), and 2mM L-glutamine (Winlab, South North Brunswick, NJ, USA). Control Culture (CC) and 17DD-YF antigen-stimulated cultures (17DD-YF Ag) were carried out by adding 100μL/well of RPMI-1640 or 17DD-YF vaccine (250LD_50_ of 17DD-YF vaccine/well). Cultures were maintained for 144 hours at 37°C in a 5% CO_2_ humidified atmosphere. Cultures were re-incubated for 4h with Brefeldin-A at 0.01mg/mL (Sigma, St Louis, MO, USA) and treated for 15min at room temperature with 2mM ethylenediamine-tetra-acetic acid—EDTA (Sigma, St Louis, MO, USA). Cells were harvested and washed twice with FACS buffer (Phosphate-buffered saline—PBS, supplemented with 0.5% bovine serum albumin, 0.1% sodium azide, Sigma, St Louis, MO, USA). The cell suspension was adjusted to 1x10^6^ cells/mL with FACS buffer.

### Analysis of 17DD-YF memory-related phenotypic biomarkers—T and B-cell surface molecules

After 17DD-YF antigen recall *in vitro*, the cell suspension (1x10^6^ cells/mL) was stained with Live/Dead Dye (Life Technologies, Carlsbad, CA, USA) and incubated with a cocktail of monoclonal antibodies anti-cell surface molecules to identify the memory status of T and B-cell subsets, comprising of: FITC/anti-CD4/(RPA-T4), PerCP-Cy5.5/anti-CD8/(SK1), PE/anti-CD27/(M-T271) and PE-Cy7/anti-CD45RO/(UCHL1) and APC-Cy7/anti-CD3/(SK7) for T-cell immunostaining and FITC/anti-IgD/(IA6-2), PE/anti-CD27/(M-T271) and PerCP/anti-CD19/(HIB19) for B-cell analysis, all from BD Pharmingen (BD Bioscience, San Diego, CA, USA). Stained cells were washed once with FACS buffer, fixed with of FACS fixing solution (10g/L paraformaldehyde, 10.2g/L sodium cacodylate and 6.63g/L sodium chloride, pH 7.2) and stored at 4°C prior acquisition on a BD LSR Fortessa Flow Cytometer (BD Bioscience, San Diego, CA, USA).

### Analysis of 17DD-YF memory-related functional biomarkers—Intracytoplasmic vytokine pattern

The analysis of 17DD-YF memory-related functional biomarkers was performed by intracytoplasmic cytokine staining by flow cytometry as described previously [15] and modified as follows: aliquots of 1x10^6^ cells, obtained after 17DD-YF antigen recall *in vitro*, were stained by Live/Dead Dye (Life Technologies, Life Technologies, Carlsbad, CA, USA) and incubated with anti-human monoclonal antibodies, including: Qdot605/anti-CD3/(UCHT1, Invitrogen, Carlsbad, CA, USA), APCe-Fluor780/anti-CD4/(GK1.5, eBioscience, San Diego, CA, USA), PerCP/anti-CD8/(SK1, BD Biosciences, San Diego, CA, USA) and Alexa-Fluor700/anti-CD19/(HIB19, eBioscience, San Diego, CA, USA). Stained samples were washed once with FACS buffer and resuspended in FACS lysing solution, containing paraformaldehyde, for pre-fixation. The cells were washed with FACS buffer and resuspended in FACS permeabilizing solution (FACS buffer supplemented with 0.5% saponin). Cell suspension was further stained with a cocktail of monoclonal antibodies, comprising of: Alexa-Fluor488/anti-IFN-γ/(clone B27), PE/anti-IL-5/(clone JES1-39D10), APC/anti-IL-10/(clone JES3-19F1) and PE-Cy7/anti-TNF-α/(clone MAb11) all purchased from BD Bioscience (San Diego, CA, USA). Stained cells were washed once with FACS permeabilizing buffer and then, with FACS buffer prior fixation with FACS fixing solution. Cells were stored at 4°C in the dark until acquisition on a BD LSR Fortessa Flow Cytometer (BD Bioscience, San Diego, CA, USA).

### Flow cytometric acquisition and analysis

BD LSR Fortessa flow cytometer (Becton Dickinson, San Jose, CA, USA) and the FACS DIVA software (BD Biosciences, San Jose, CA, USA) were used for data acquisition. A minimum of 100,000 events gated on lymphocytes were acquired per sample. Distinct offline gating strategies were applied to quantify the memory-related biomarkers on T and B-cells as previously described [6]. The FlowJo software version 9.3.2 was used for data analysis (TreeStar, San Diego, CA, USA). The memory T-cell subsets were quantified as the percentage of Naïve/(N)/CD27^+^CD45RO^-^; early Effector Memory/(eEf)/CD27^-^CD45RO^-^; Central Memory/(CM)CD27^+^CD45RO^+^; Effector Memory/(EM)/CD27^-^CD45RO^+^ amongst CD4^+^ and CD8^+^ T-cells. The memory B-cell subsets were quantified as the percentage of Naïve/(N)/CD27^-^IgD^+^; Non-classical Memory/(nCM)/CD27^+^IgD^+^; Classical Memory/(CM)/CD27^+^IgD^-^ within CD19^+^ B-cells. Moreover, the percentage of cytokine^+^ cells were also quantified amongst CD4^+^ and CD8^+^ T-cells (TNF-α, IFN-γ, IL-10 and IL-5) as well as CD19^+^ B-cells (TNF-α, IL-10 and IL-5).

### Data mining and analysis

Data analysis of biomarkers was performed by first converting the flow cytometric original data, expressed as percentage of memory T-cell and B-cell as well as cytokine^+^ T and B-cells into 17DD-YF Ag/CC Index, calculated as the ratio between the frequency of positive cells observed in the 17DD-YF stimulated culture (17DD-YF Ag) by the respective control culture (CC). Biomarker signature analysis was carried out using the global median value of 17DD-YF Ag/CC Index for each biomarker was used as the cut-off edge to define low or high 17DD-YF Ag/CC Index. The memory-related biomarker signatures of PV(day30-45) group was employed as the reference curve for comparative analysis amongst the other study groups. Those biomarkers with more than 50% of volunteers above the cut-off index were selected for further analysis amongst groups. Substantial change in the set of relevant biomarkers were highlighted by (*), when the proportion of subjects above the cut-off fell below 50%. The common set of relevant biomarkers in each study group was underscored in bold font. This binary approach, using the median of each marker and comparing the proportion of subjects with high values to the proportion observed in PV(day30-45), allows the identification of slight differences in proportion of even when the overall changes in biomarker levels are subtle. Additional analysis was carried out employing Venn diagram (http://bioinformatics.psb.ugent.be/webtools/Venn/), ROC curve (MedCalc software package, Version 7.3.0.0), heatmap (R Project for Statistical Computing Version 3.0.1) and decision tree J48 algorithm (present in WEKA software version 3.6.11). The J48 is an implementation of C4.5 algorithm (insert citation #1) for inducing classification decision trees. The classification task is the main approach of data mining and it aims at identifying the attribute set, found in the dataset, that classifies all samples of dataset with the best accuracy. Decision tree classifiers execute supervised learning and balancing good accuracy as well as simple knowledge representation. The J48 method provides ten (10) parameters, such as: binarySplits, confidenceFactor, minNumObj, numFolds, reducedErrorPruning, saveInstanceData, seed, subtreeRaising, unprunned and useLaplace. The best results were found using default parameters. The goal of using the decision tree algorithm was to identify the set of phenotypic and functional attributes of YF-specific immunity that could support the hypothesis that, while PV(day30-45) & PV(year1-9) exhibited relevant YF-specific biomarkers, NV (day0) & PV(year10-11) did not present such relevant potential correlates of protection. Once defined the root attributes for the decision trees of phenotypic memory and functional analysis, the software was run again using only the root attributes. Leave-one-out-cross-validation analysis (LOOCV) was employed to minimize biased performance estimates by using all data set for decision tree model fitting. Additionally, Chi-square test and Spearman’s correlation test were calculated using Prism GraphPad software 5.03.

## Results

### Overall 17DD-YF memory-related biomarker signatures at distinct time-points after primary vaccination

**Fig 1:**
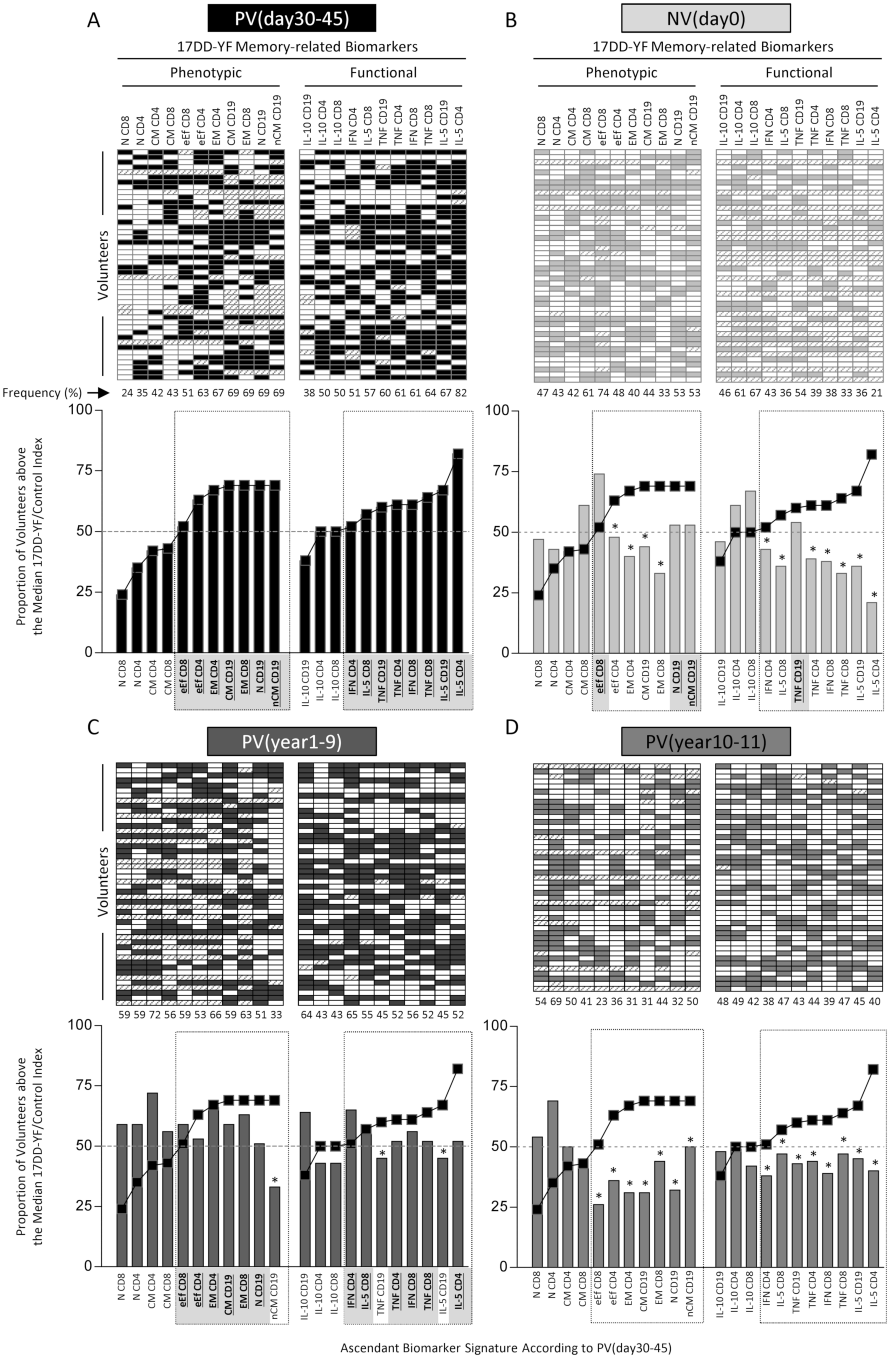
Overall 17DD-YF memory-related biomarker signatures at distinct time-points after primary vaccination. The phenotypic/functional biomarker signatures were built taking the proportion of subjects above the cut-off edges defined for each attribute, calculated as the median index value (17DD-YF/Control) for the study population. Diagrams were constructed for all study groups to calculate the proportion (%) of volunteers above the median cut-off indices for each biomarker (gray-shaded spots). (A) The PV(day30-45) group was used to construct the memory-related phenotypic and functional biomarker signatures and draw the reference curves, used for comparative analysis amongst the study groups, (B) NV(day0), (C) PV(year1-9) and (D) PV(year10-11). Hatched cells represent unavailable results. Data mining was carried out as proposed previously by Luiza-Silva et al., (2011), selecting from the PV(day30-45) reference curves, those biomarkers with more than 50% of volunteers above the cut-off index (surrounded by dashed rectangles). Comparative analysis amongst the study groups were carried out considering only the selected set of relevant biomarkers from the phenotypic and functional reference curves. Substantial change in the set of relevant biomarkers were highlighted by (*) when the proportion of subjects above the cut-off fell below 50%. The common set of relevant biomarkers on each study group was underscored in bold font.

The phenotypic/functional biomarker signatures are presented in Fig 1. Gray-shaded diagrams were assembled for all study groups to determine the proportion (%) of volunteers above the median cut-off indices for each study. The biomarker signatures of PV(day30-45) were used to select a set of seven phenotypic (eEfCD8;eEfCD4;EMCD4; CMCD19;EMCD8;NCD19;nCMCD19) and eight functional memory-related biomarkers (IFNCD4;IL-5CD8;TNFCD19;TNFCD4;IFNCD8;TNFCD8;IL-5CD19;IL-5CD4) was predominant in the biomarker signatures of the PV(day30-45) (Fig 1A—dashed rectangles). Comparative analysis demonstrated that NV(day0) displayed a clear deficiency on a range of memory-related biomarkers as compared to PV(day30-45), except eEfCD8, NCD19, nCMCD19 and TNFCD19 (Fig 1B—dashed rectangles). The PV(year1-9) group presented most memory-related biomarkers identified above the 50^th^ percentile in the PV(day30-45) group, except nCMCD19, TNFCD19 and IL-5CD19 (Fig 1C—dashed rectangles). It was clearly observed that PV(year10-11) lost all memory-related biomarkers as compared to PV(day30-45) (Fig 1D—dashed rectangles).

### 17DD-YF memory-related biomarker signatures according to the age at primary vaccination

**Fig 2:**
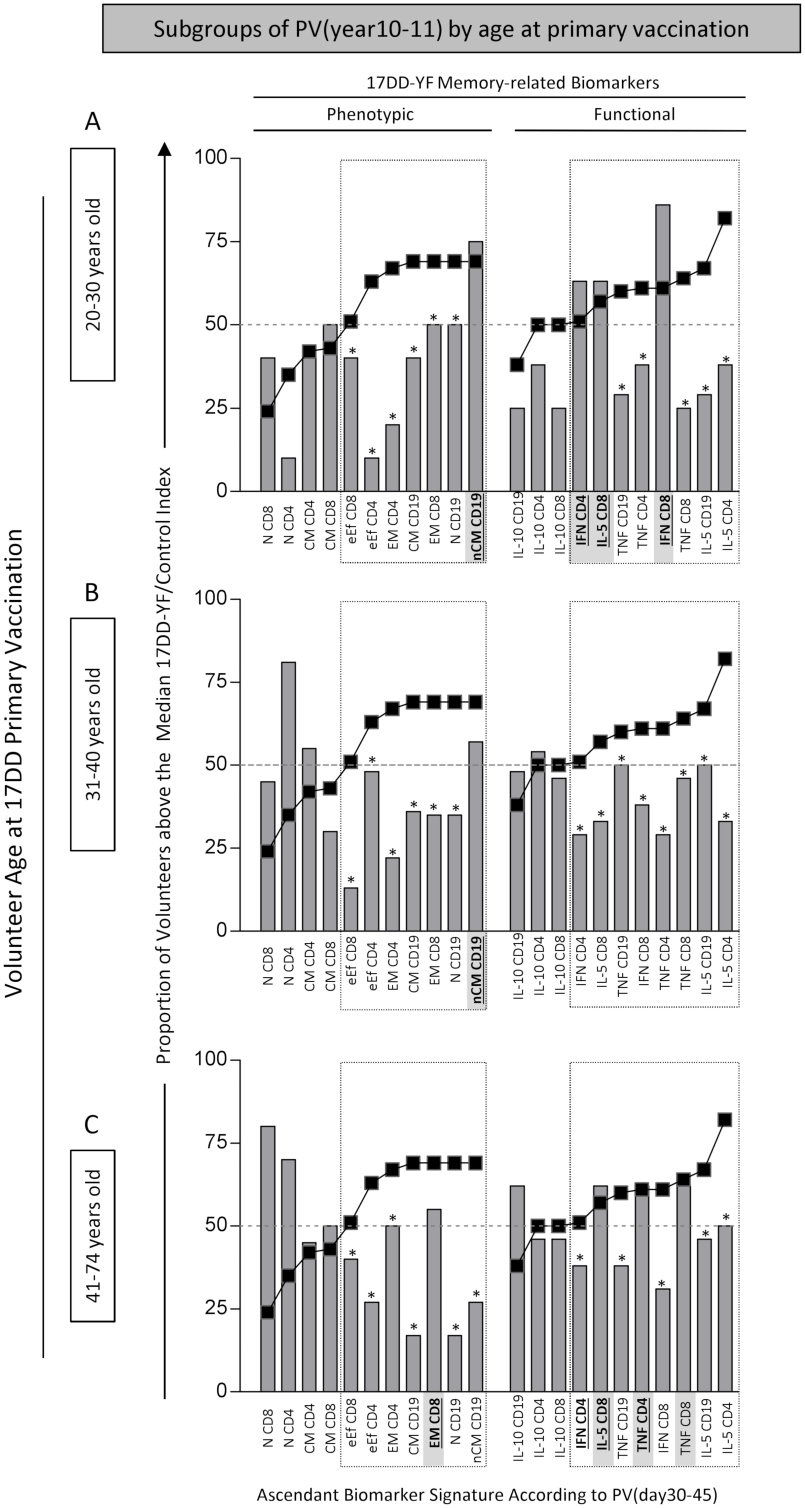
17DD-YF memory-related biomarker signatures according to the age at primary vaccination. The memory-related biomarker signatures were constructed using the proportion of subjects above the cut-off edges defined for each attribute, calculated as the median index value (17DD-YF/Control) for the study population. The signatures were constructed for phenotypic/functional biomarkers, compiling the proportion (%) of volunteer above the median cut-off indices. The memory-related biomarker signatures, constructed for the PV(day30-45) group, were used as the reference curves for comparative analysis amongst the PV(year10-11) subgroups, categorized according to the age at primary vaccination, including (A) 20–30 years old, (B) 31–40 years old and (C) 41–74 years old. Comparative analysis were carried out considering only the set of relevant biomarkers pre-selected from the phenotypic/functional reference curves (surrounded by dashed rectangles), including those biomarkers with more than 50% of volunteers above the cut-off index in the PV(day30-45) reference curves (surrounded by dashed rectangles). Substantial changes in the set of relevant biomarkers were underscored by (*) when the proportion of subjects above the cut-off fell below 50%. The common set of relevant biomarkers on each study group was underscored in bold font.

To determine whether the loss of all memory-related biomarkers observed in PV(year10-11) was somehow influenced by the age at 17DD-YF primary vaccination, the memory-related biomarker signatures were evaluated in subgroups of PV(year10-11) referred to as: young adults (20–30 years old—Fig 2A), middle age (31–40 years old—Fig 2B) and older adults (41–74 years old—Fig 2C) Data analysis demonstrated that regardless of the age at 17DD-YF primary vaccination, a significant drop in memory-related biomarker could be observed 10–11 years after primary vaccination.

### Set of phenotypic/functional biomarkers useful to monitor the memory status following 17DD-YF primary vaccination

**Fig 3:**
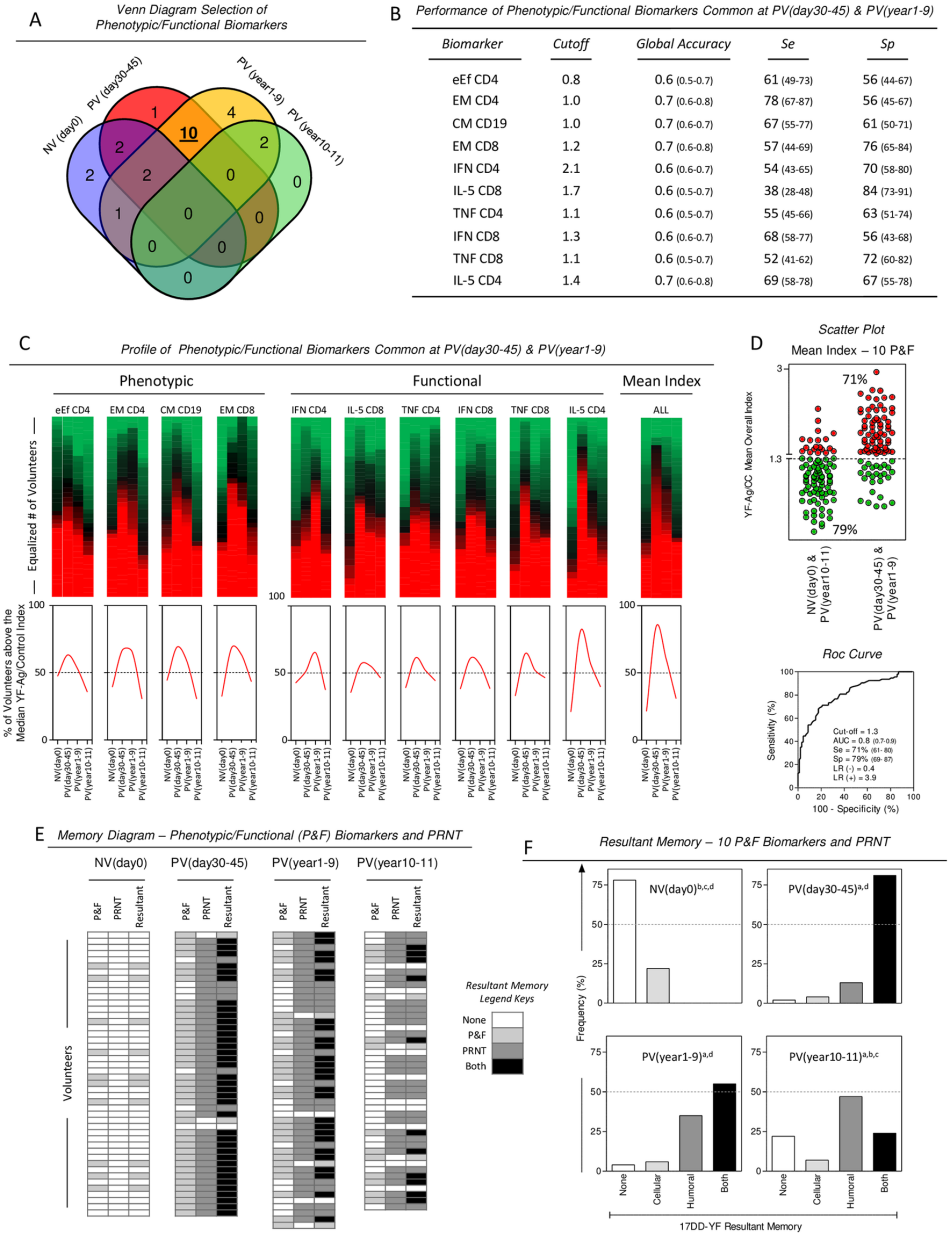
Set of phenotypic/functional biomarkers useful to monitor the memory status following primary 17DD-YF vaccination. (A) Venn diagram analysis was carried out to identify the intersection of phenotypic/functional biomarkers amongst NV(day0), PV(day30-45), PV(year1-9) and PV(year10-11) and select those common attributes between PV(day30-45) and PV(year1-9), considered biomarkers of protection. Detailed Venn Diagram report is provided in the S1 Table. (B) The performance indicators (cut-off, accuracy, sensitivity-Se and specificity-Sp) for the ten selected common biomarkers (eEfCD4, EMCD4, CMCD19, EMCD8, IFNCD4, IL-5CD8, TNFCD4, IFNCD8, TNFCD8 and IL-5CD4) are provided in the inserted table. (C) Heatmap analysis was carried out to illustrate the profile of phenotypic/functional biomarkers common at PV(day30-45) and PV(year1-9) as well as the mean index calculated considering all 10 phenotypic/functional biomarkers together and demonstrate the proportion (%) of volunteers presenting low (Green), median (Black) or high (Red) YF-Ag/CC index. Histograms were constructed to highlight the frequency of volunteers above the median YF-Ag/CC index (Red Curves). (D) Scatter plot were built to demonstrate the sensitivity (Red Circles) and specificity (Green Circles) of the mean index of 10 phenotypic/functional biomarkers, using the cut-off edge (Mean Index = 1.3) provided by the ROC curve analysis. (E) Memory diagram were constructed using the defined cut-off for phenotypic/functional biomarkers, the memory status was defined for each subject, considering the phenotypic/functional (Mean Index of 10 biomarkers >1.3) and PRNT (>2.9 Log mIU/mL, according to Simões et al., 2012). Column statistics were used to calculate the proportion of subjects displaying distinct status of resultant memory, referred as none, phenotypic/functional biomarkers—P&F, PRNT and both. (F) The resultant memory for the 10 phenotypic/functional biomarkers and PRNT was displayed on bar charts. Significant differences at p<0.05 (Chi-square test) of resultant status amongst study groups were represented by letters “a”, b”, “c” and “d” in comparison to NV(day0), PV(day30-45), PV(year1-9) and PV(year10-11), respectively.

The Venn diagram was generate using all possible logical relations between a finite collection of different biomarker sets including those presenting frequency above the 50^th^ percentile for each group (Fig 1 –dashed rectangle selections). Venn diagram analysis demonstrated that 10 out of 22 biomarkers (eEfCD4;EMCD4;CMCD19;EMCD8;IFNCD4;IL-5CD8;TNFCD4; IFNCD8;TNFCD8;IL-5CD4) were commonly present in more than 50% of volunteers in PV(day30-45) & PV(year1-9)and were considered potential correlates of protection (Fig 3A). Detailed Venn diagram report is provided in the S1 Table. The performance indices revealed that although EMCD4, CMCD19, EMCD8 and IL-5CD4 are the most promising biomarkers, individually, they presented low global accuracy (AUC = 0.7) [15] (Fig 3B).

Heatmap assemblage was constructed for each selected biomarker and also for the overall mean of the 10 biomarkers taken together (Fig 3C). The performance analysis of the overall mean enhanced the global accuracy (AUC = 0.8) [15], with concomitant increases in sensitivity/(Se = 71%) and specificity/(Sp = 79%) (Fig 3D).

The overall mean of 10 biomarkers were further used to calculate the resultant memory status alongside with the PRNT titers. A memory biomarker diagram was assembled to compile the categorical data of phenotypic/functional attributes (overall mean >1.3) and PRNT (>2.9mIU/mL) for each volunteer and calculate the resultant memory status, referred as: None, phenotypic/functional (P&F), PRNT or both (P&F and PRNT) (Fig 3E). Data analysis demonstrated that in the NV(day0) group, there was a predominant proportion (78%) of subjects with no memory attributes (None), whereas in the PV(day30-45) a clear majority of volunteers (81%) presented both memory attributes. In PV(year1-9) there was a sustained prevalence of volunteers with both memory attributes (55%). However, in the PV(year10-11) a clear drop on the frequency of volunteers with both memory attributes (24%) was observed. It was evident that over 20% of vaccinees lack either memory attribute, 10–11 years after 17DD-YF primary vaccination (Fig 3F).

### Major phenotypic/functional biomarkers useful to monitor the memory status following 17DD-YF primary vaccination

**Fig 4:**
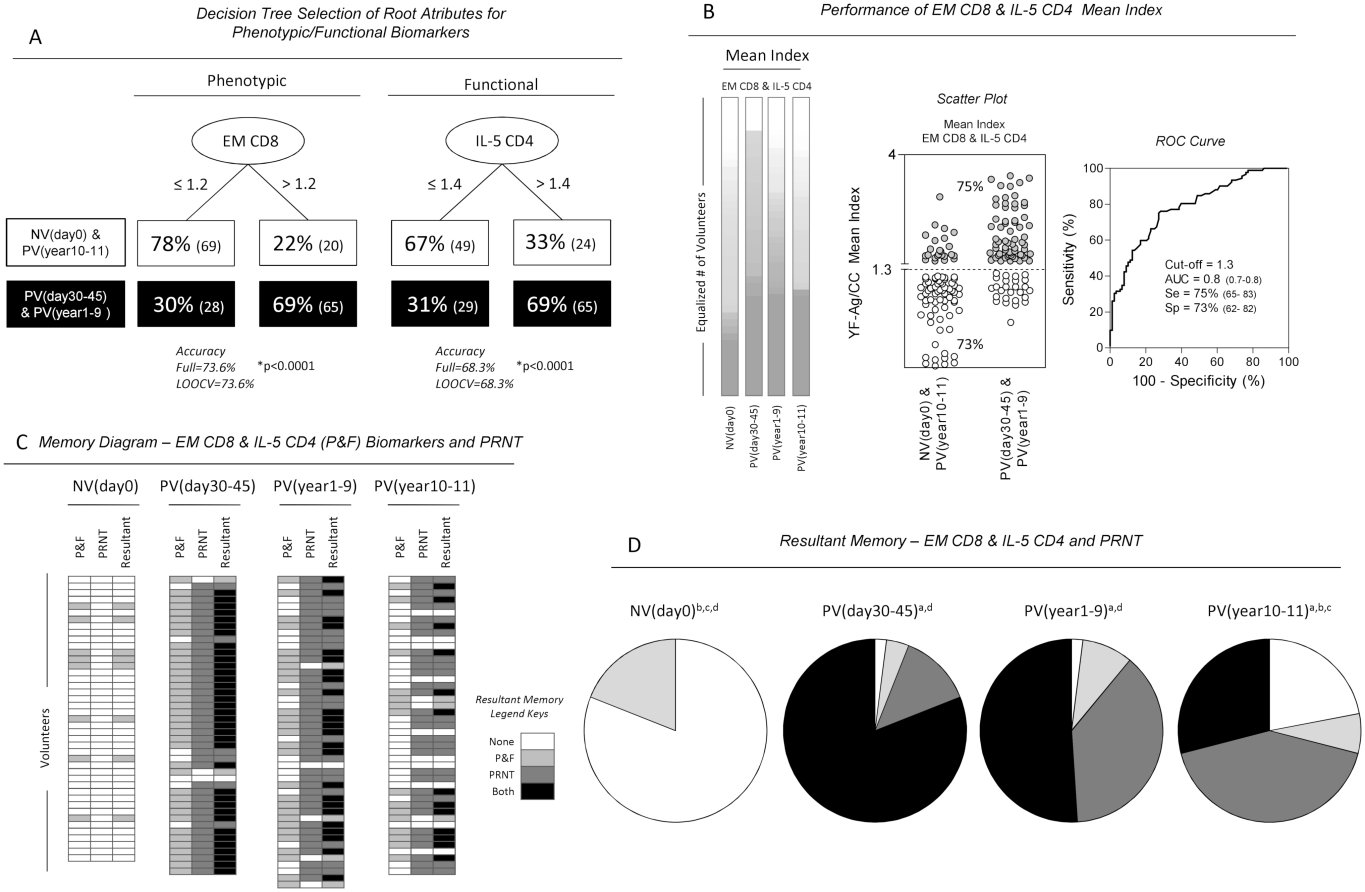
Major phenotypic/functional biomarkers useful to monitor the memory status following primary 17DD-YF vaccination. (A) Decision tree analysis was carried out to identify root attributes [Ellipses] for phenotypic/functional (P&F) biomarkers amongst NV(day0)&PV(year10-11) [white rectangle] and PV(day30-45)&PV(year1-9) [black rectangle], considered biomarkers to discriminate unprotected from protected subjects. Leave-one-out-cross-validation analysis (LOOCV) was employed to minimize biased performance estimates by using all data set for decision tree model fitting. EMCD8 and IL-5CD4 were selected as major phenotypic and functional 17DD-YF Memory-related biomarkers, respectively. (B) Heatmaps were built, taking the mean index of the top-two phenotypic/functional biomarkers (EMCD8 & IL-5CD4) and demonstrating the proportion (%) of volunteers ranging from low (White) to high (Gray) YF-Ag/CC index. A scatter plot was constructed to show the sensitivity (Gray Circle) and specificity (White Circle) of the top-two biomarkers, employing the cut-off edge (Mean Index = 1.3) provided by the ROC curve analysis. (C) Resultant memory status was defined for each subject, considering the top-two biomarkers (Mean Index >1.3) and PRNT (>2.9 Log mIU/mL, according to Simões et al., 2012). Column statistics were used to calculate the proportion of subjects displaying differing categories of resultant memory, referred as none, top-two biomarkers—P&F, PRNT and both. (D) Pie charts illustrated the overall resultant memory status within each category, as determined by the top-two biomarkers and PRNT. Significant differences at p<0.05 (Chi-square test) of resultant memory status amongst study groups were represented by letters “a”, b”, “c” and “d” in comparison to NV(day0), PV(day30-45), PV(year1-9) and PV(year10-11), respectively.

Two independent decision tree algorithms were generated using a set of selected features provided by the Venn diagram. The attributes included in these models of decision tree algorithms were the following: (eEfCD4;EMCD4;CMCD19;EMCD8) for phenotypic memory assessment and (IFNCD4;IL-5CD8;TNFCD4;IFNCD8;TNFCD8;IL-5CD4) for functional analysis. Data analysis pointed out that EMCD8 and IL-5CD4 were the top-two phenotypic and functional root attributes able to segregate PV(day30-45) & PV(year1-9) from NV(day0) & PV(year10-11) (Fig 4A). The rational to use this approach was the hypothesis that NV (day0) & PV(year10-11) displayed a relevant lacks of YF-specific memory biomarkers while PV(day30-45) & PV(year1-9) exhibited most of the memory biomarkers considered relevant potential correlates of protection. Using this approach the decision tree employing the selected root biomarkers, as independent attributes, yielded a classification of 78% and 67% of NV(day0) & PV(year10-11) with EMCD8 and IL-5CD4 below the cut-off point while 69% of PV(day30-45) & PV(year1-9) presented these biomarkers above the cut-off points. The accuracy of EMCD8 and IL-5CD4 employed as independent attribute was 0.74 and 0.68, respectively (Fig 4A).

Considering the moderate accuracy of these attributes employed as independent attributes, the combination of EMCD8 & IL-5CD4 were proposed, which led to increased performance (AUC = 0.8, Se = 75%, Sp = 73%) as compared to the use of a single attribute (Fig 4B).

The overall values for EMCD8 & IL-5CD4 Mean Index were further used to calculate the resultant memory status alongside the PRNT titers. A memory biomarker diagram was constructed using the categorical data of EMCD8 & IL-5CD4 (overall mean >1.3) and PRNT (>2.9mIU/mL) and calculate the resultant memory status (Fig 4C). The results demonstrated that while NV(day0) group presented a major proportion (81%) of subjects with no memory attributes (None), the PV(day30-45) displayed a predominance (80%) of volunteers with both memory attributes. The PV(year1-9) presented a persistent prevalence of volunteers with both memory attributes(51%). The PV(year10-11) showed a conspicuous decrease in the proportion of volunteers with both memory attributes (29%). Noteworthy was that over 22% of these vaccinees lack the top-two phenotypic/functional biomarkers and the PRNT memory attributes (Fig 4D).

### Changes in neutralizing antibody titers and phenotypic/functional memory-related biomarkers at distinct time-points after 17DD-YF primary vaccination

**Fig 5:**
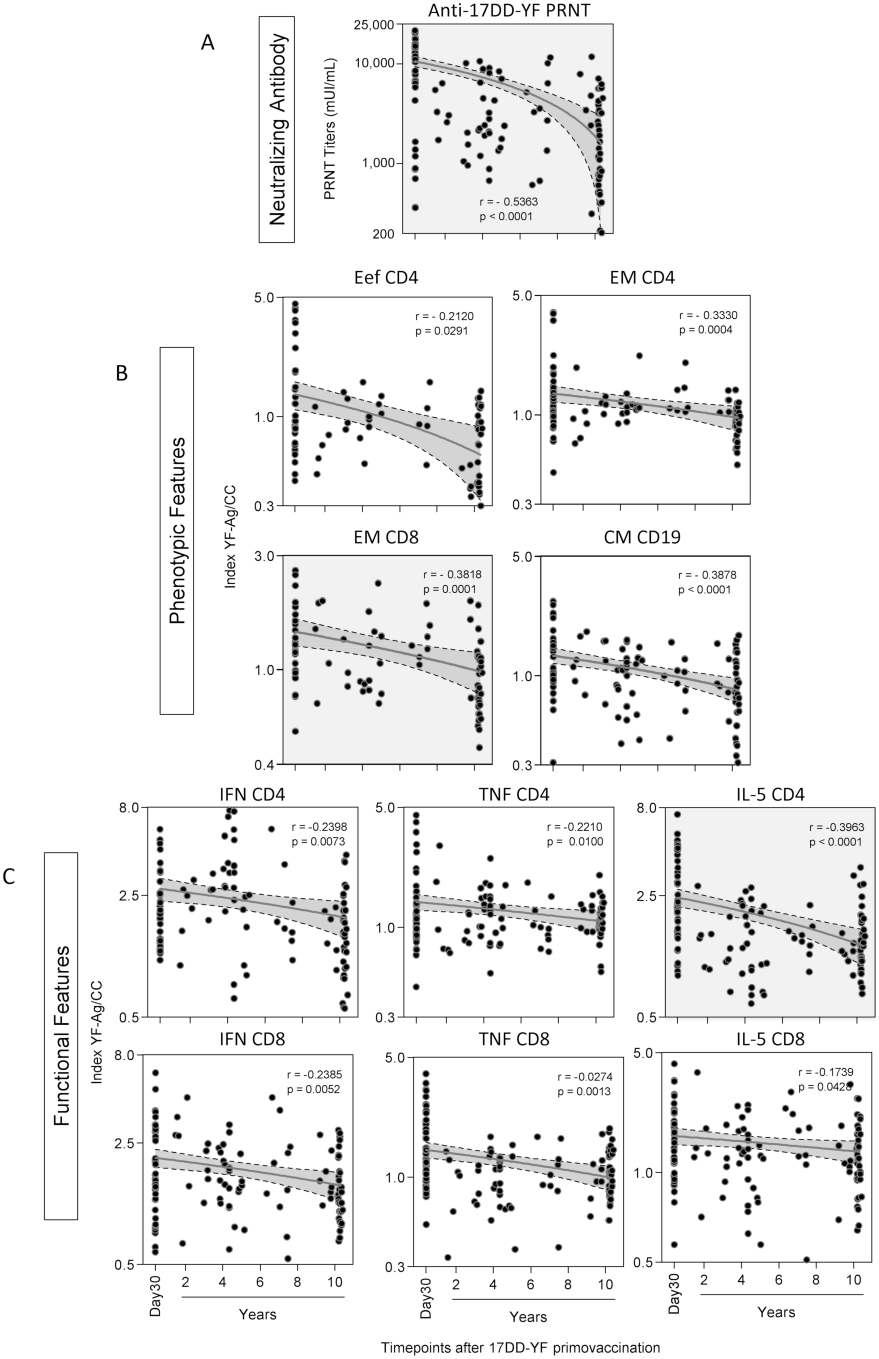
Changes in neutralizing antibody titers and phenotypic/functional memory-related biomarkers at distinct time-points after primary 17DD-YF vaccination. Correlation analyses were performed to validate the time-dependent decline in (A) neutralizing antibody titers and 17DD-YF Memory-related (B) phenotypic and (C) functional features. Data are expressed as scattering distribution of individual values along distinct time-points after 17DD-YF primary vaccination against neutralizing antibody titers (PRNT) as well as phenotypic and functional features (YF-Ag/CC Index). Spearman’s correlation test was applied to identify significant time-dependent loss of memory-related biomarkers. Correlation indices (p and r) along with the 95% confidence band of the best-fit line are provided in the figure. Attributes with higher correlation indices (r values) are highlighted with gray background.

Spearman’s correlation analysis was carried out to determine whether the changes in PRNT titers as well as phenotypic/functional memory-related features observed after 17DD-YF primary vaccination exhibit a continuously decreasing profile over time (Fig 5). The results demonstrated a time-dependent decrease in neutralizing antibody titers (PRNT) (Fig 5A) as well as phenotypic (Fig 5B) and functional features (Fig 5C). The correlation indices also confirmed that the top-two biomarkers (EMCD8 and IL-5CD4) presented the higher correlation indices (Fig 5B and 5C—gray background).

## Discussion

The present study have two major goals: i) broaden the set of biomarkers available for monitoring of post-vaccination YF-specific immunity and ii) define the bet set of biomarkers to identify individuals considered not protected. For this purpose, we have performed a cross-sectional investigation including volunteers living in endemic areas for YF categorized according to the time after 17DD-YF vaccination that consisted of unvaccinated subjects [NV(day0)] and three groups of primary vaccinated volunteers [PV(day30-45), PV(year1-9) and PV(year10-11)]. In this sense, the inclusion of non-vaccinated subjects and primary vaccinated individuals early after vaccination was the rational to first identify the best set of biomakers for post-vacination monitoring purposes. Once identified the biomarkers that could be referred as potential correlates of protection, i.e. those observed in PV(day30-45) but not in NV(day0), the perfomance of each biomarker was evaluated and those with better acuray employed to monitor the immune response along time to search for putative decline in the YF-specific immunity.

The characterization of the YF-specific memory overtime after primary vaccination would provide insights that underscore the conspicuous time-dependent decrease of resultant memory following 17DD-YF primary vaccination that might be relevant to maintain the protection in areas under risk of YF transmission. Machine-learning techniques were employed to select the most suitable biomarkers to perform a qualitative and quantitative analysis of cellular and humoral immunity following the 17DD-YF primary vaccination.

The comparative analysis demonstrated that the primary vaccination triggers in PV(day30-45) an increase of several phenotypic and functional memory-related biomarkers as compared to NV(day0). This set of biomarkers included seven phenotypic (eEfCD8;eEfCD4;EMCD4;CMCD19;EMCD8;NCD19;nCMCD19) and eight functional memory-related biomarkers (IFNCD4;IL-5CD8;TNFCD19;TNFCD4;IFNCD8;TNFCD8;IL-5CD19;IL-5CD4). Our findings corroborate previous data from longitudinal studies that report an increase of memory CD8^+^ T-cells cells after 17D-YF vaccination [16,17]. Expansion of memory CD8^+^ T-cells (CCR7^-^, Ki-67^-^ and Bcl-2^+^) has been reported following vaccination 17D-YF vaccination as a gradual and continuous process associated with high-quality immune memory observed at early (3 month) and late (2 years) stages [16]. Increase of effector CD8^+^ T-cells (PD-1^+^, CCR7^-^ and CD45RA^-^) after 17D-YF primary vaccination have also been reported early at day15. Moreover, these authors have demonstrated that regardless the polyfunctional profile of CD8^+^ T-cells, the positivity for IFN-γ was patent at day10, day15 and day90 [18]. The increase of IL-5 produced by CD4^+^ and CD8^+^ T-cells has been already reported at day30 after 17DD-YF primary vaccination [2].

Most of those biomarkers identified in PV(day30-45) remained consistently present in PV(year1-9), but a clear loss of magnitude of all memory-related biomarkers was observed in PV(year10-11). The decrease in memory-related biomarker observed in PV(year10-11)was not influenced by the age at primary vaccination. Previous studies have in fact demonstrated that age did not affect the serum neutralization index after 17D-YF primary vaccination [18].

Studies addressing the decline overtime of the complex immune response elicited by the YF-vaccines have previously demonstrated that neutralizing antibodies as well as the YF-specific T-cells response display a time-dependent decrease following primary vaccination [4,6,12,13]. It has been demonstrated that the PRNT titers, considered the “gold standard” for correlate of protection following YF vaccination decline overtime [4]. Decrease of T and B-cell together with lower PRNT titers has already been reported [6,19]. The reduced persistence of cellular and humoral memory in vaccinated subjects, suggestive of a waning of potential correlates of protection over time, is a relevant aspect that should be considered to support the guidelines of revaccination in areas under risk of YF transmission.

In the present investigation, we intended to select promising phenotypic and functional memory-related biomarkers to monitor, beyond the PRNT assays, the duration of vaccination-induced 17DD-YF memory. Our finding initially pointed out 10 biomarkers as putative potential correlates of protection commonly observed in PV(day30-45) and PV(year1-9), including:(eEfCD4;EMCD4;CMCD19;EMCD8;IFNCD4;IL5CD8;TNFCD4;IFNCD8;TNFCD8;IL-5CD4). From these biomarkers, (EMCD8;IL-5CD4) were elected as the top-two biomarkers to be used, along with the PRNT, to monitor the duration protective memory following the 17DD-YF vaccination. Using this selected set of biomarkers to define the resultant memory our finding demonstrated conspicuous decrease of resultant memory along with the complete lack of memory-related attributes found in 22% of volunteers from PV(year10-11). Whether the decrease in the selected biomarkers leads to loss of protection cannot be addressed in this study. However, these laboratorial findings should not be neglected since it provides evidence of the concomitant decrease of PRNT seropositivity and YF-specific cellular immunity over time, reaching critical values at 10 years after 17DD-YF primary vaccination. The decline of PRNT seropositivity and cellular immunity biomarkers over time upon primary vaccination does not imply in the lack of vaccine efficacy but provides important information for future YF vaccine research. It is important to mention that this study did not examine the impact of selection bias such as gender, age and their possible influence on the memory biomarker profile observed. Moreover, the global accuracy and the leave-one-out cross-validation approaches provided by these biomarkers were calculated employing only data used to develop the model of decision tree algorithm, with no additional validation set. Additionally, this study may have limitations for using study-specific global cutoffs that require further confirmation for field applications in multiple studies.

In conclusion, our findings ultimately demonstrated that the simultaneous analysis of YF-specific cellular and humoral immunity using the selected biomarkers (EMCD8;IL5CD4;PRNT) is a useful tool to monitor the YF-vaccine responses and memory persistence in distinct populations to define the resultant memory status. The conspicuous time-dependent decrease of resultant memory following 17DD-YF primary vaccination might be relevant to monitor potential correlates of protection in areas under risk of YF transmission.

## Supporting information

S1 Table Venn diagram report for the intersection analysis of phenotypic/functional (P&F) biomarkers amongst NV(day0), PV(day30-45), PV(year1-9) and PV(year10-11). Elements underscored and in bold correspond to biomarkers associated to protection.
(TIFF)

S1 Checklist The checklist for the cross-sectional study was fulfilled and added in the online submission system as Costa-Pereira et al STROBE checklist file. (DOC)

## References

[1] Monath TP, Cetron MS, McCarthy K, Nichols R, Archambault WT, Weld L et al Yellow fever 17D vaccine safety and immunogenicity in the elderly. *Hum*. *Vaccine*. 2005; 1: 207–214. 10.4161/hv.1.5.2221 17012867

[2] Silva ML, Martins MA, Espírito-Santo LR, Campi-Azevedo AC, Silveira-Lemos D, Ribeiro JG et al Characterization of main cytokine sources from the innate and adaptive immune responses following primary 17DD yellow fever vaccination in adults. *Vaccine*. 2011; 29: 583–592. doi: 10.1016/j.vaccine.2010.08.046 20732465 10.1016/j.vaccine.2010.08.046

[3] Ahmed R, Akondy RS. Insights into human CD8(+) T-cell memory using the yellow fever and smallpox vaccines.*Immunol Cell Biol*. 2011; 89: 340–345. doi: 10.1038/icb.2010.155 21301482 10.1038/icb.2010.155

[4] Collaborative group for studies on yellow fever vaccines. Duration of post-vaccination immunity against yellow fever in adults. *Vaccine*. 2014; 32: 4977–4984. doi: 10.1016/j.vaccine.2014.07.021 25090646 10.1016/j.vaccine.2014.07.021

[5] Campi-Azevedo AC, de Araújo-Porto LP, Luiza-Silva M, Batista MA, Martins MA, Sathler-Avelar R et al 17DD and 17D-213/77 yellow fever substrains trigger a balanced cytokine profile in primary vaccinated children. *PLoS One*. 2012; 7:e49828. 23251351 10.1371/journal.pone.0049828 PMC3519464

[6] Campi-Azevedo AC, Costa-Pereira C, Antonelli LR, Fonseca CT, Teixeira-Carvalho A, Villela-Rezende G, Santos RA et al Booster dose after 10 years is recommended following 17DD-YF primary vaccination. *Hum Vaccin Immunother*.2016; 12: 491–502. doi: 10.1080/21645515.2015.1082693 26360663 10.1080/21645515.2015.1082693 PMC5049740

[7] Martins MA, Silva ML, Marciano AP, Peruhype-Magalhães V, Eloi-Santos SM, Ribeiro jG et al Activation/modulation of adaptive immunity emerges simultaneously after 17DD yellow fever first-time vaccination: is this the key to prevent severe adverse reactions following immunization? *Clin Exp Immunol*. 2007; 148: 90–100. doi: 10.1111/j.1365-2249.2006.03317.x 17309541 10.1111/j.1365-2249.2006.03317.x PMC1868854

[8] Martins MA, Silva ML, Elói-Santos SM, Ribeiro JG, Peruhype-Magalhães V, Marciano AP et al Innate immunity phenotypic features point toward simultaneous raise of activation and modulation events following 17DD live attenuated yellow fever first-time vaccination. *Vaccine*. 2008; 26: 1173–1184. doi: 10.1016/j.vaccine.2007.12.035 18243433 10.1016/j.vaccine.2007.12.035

[9] Gaucher D, Therrien R, Kettaf N, Angermann BR, Boucher G, Filali-Mouhim A et al Yellow fever vaccine induces integrated multilineage and polyfunctional immune responses. *J Exp Med*. 2008; 205: 3119–3131. doi: 10.1084/jem.20082292 19047440 10.1084/jem.20082292 PMC2605227

[10] Monath TP. Treatment of yellow fever. *Antiviral Res*. 2008; 78: 116–124. doi: 10.1016/j.antiviral.2007.10.009 18061688 10.1016/j.antiviral.2007.10.009

[11] Wieten RW, Jonker EF, van Leeuwen EM, Remmerswaal EB, Ten Berge IJ, de Visser AW et al A Single 17D Yellow Fever Vaccination Provides Lifelong Immunity; Characterization of Yellow-Fever-Specific Neutralizing Antibody and T-Cell Responses after Vaccination. *PLoS One*. 2016; 11: e0149871 doi: 10.1371/journal.pone.0149871 26977808 10.1371/journal.pone.0149871 PMC4792480

[12] Niedrig M, Stolte N, Fuchs D, Hunsmann G, Stahl-Hennig C. Intranasal infection of macaques with yellow fever (YF) vaccine strain 17D: a novel and economical approach for YF vaccination in man.*Vaccine* 1999; 17: 1206–1210. 10195634 10.1016/s0264-410x(98)00344-2

[13] Poland JD, Calisher CH, Monath TP, Downs WG, Murphy K. Persistence of neutralizing antibody 30–35 years after immunization with 17D yellow fever vaccine. *Bull World Health Organ*. 1981; 59: 895–900. 6978196 PMC2396120

[14] Simões M, Camacho LAB, Yamamura AMY, Miranda EH, Cajaraville ACRA, Freire MS. Evaluation of accuracy and reliability of the plaque reduction neutralization test (micro-PRNT) in detection of yellow fever virus antibodies. *Biologicals*. 2012; 40: 399–404. doi: 10.1016/j.biologicals.2012.09.005 23034357 10.1016/j.biologicals.2012.09.005

[15] Swets JA Measuring the accuracy of diagnostic systems. Science. 1988; 240: 1285–1293. 3287615 10.1126/science.3287615

[16] Akondy RS, Monson ND, Miller JD, Edupuganti S, Teuwen D, Wu H et al The yellow fever virus vaccine induces a broad and polyfunctional human memory CD8+ T cell response. *J Immunol*. 2009; 183: 7919–7930. doi: 10.4049/jimmunol.0803903 19933869 10.4049/jimmunol.0803903 PMC3374958

[17] Blom K, Braun M, Ivarsson MA, Gonzalez VD, Falconer K, Moll M et al Temporal dynamics of the primary human T cell response to yellow fever virus 17D as it matures from an effector- to a memory-type response. *J Immunol*. 2013; 190: 2150–2158. doi: 10.4049/jimmunol.1202234 23338234 10.4049/jimmunol.1202234

[18] Monath TP, Nichols R, Archambault WT, Moore L, Marchesani R, Tian J et al Comparative safety and immunogenicity of two yellow fever 17D vaccines (ARILVAX and YF-VAX) in a III multicenter, double-blind clinical trial. *Am*. *J*. *Trop*. *Med*. *Hyg*. 2002; 66: 533–541. 12201587 10.4269/ajtmh.2002.66.533

[19] Muyanja E, Semaganda A, Ngauv P, Cubas R, Perrin H, Srinivasan D et al Immune activation alters cellular and humoral responses to yellow fever 17D vaccine. *J Clin Invest*. 2014; 124: 3147–3158. doi: 10.1172/JCI75429 24911151 10.1172/JCI75429 PMC4071376

