# Supplementary figures and images for: Multi-parameter approach to evaluate the timing of memory status after 17DD-YF primary vaccination
Source: PLoS Negl Trop Dis. 2018 Jun 7;12(6):e0006462. doi: 10.1371/journal.pntd.0006462 (PMC5991646; doi:10.1371/journal.pntd.0006462)

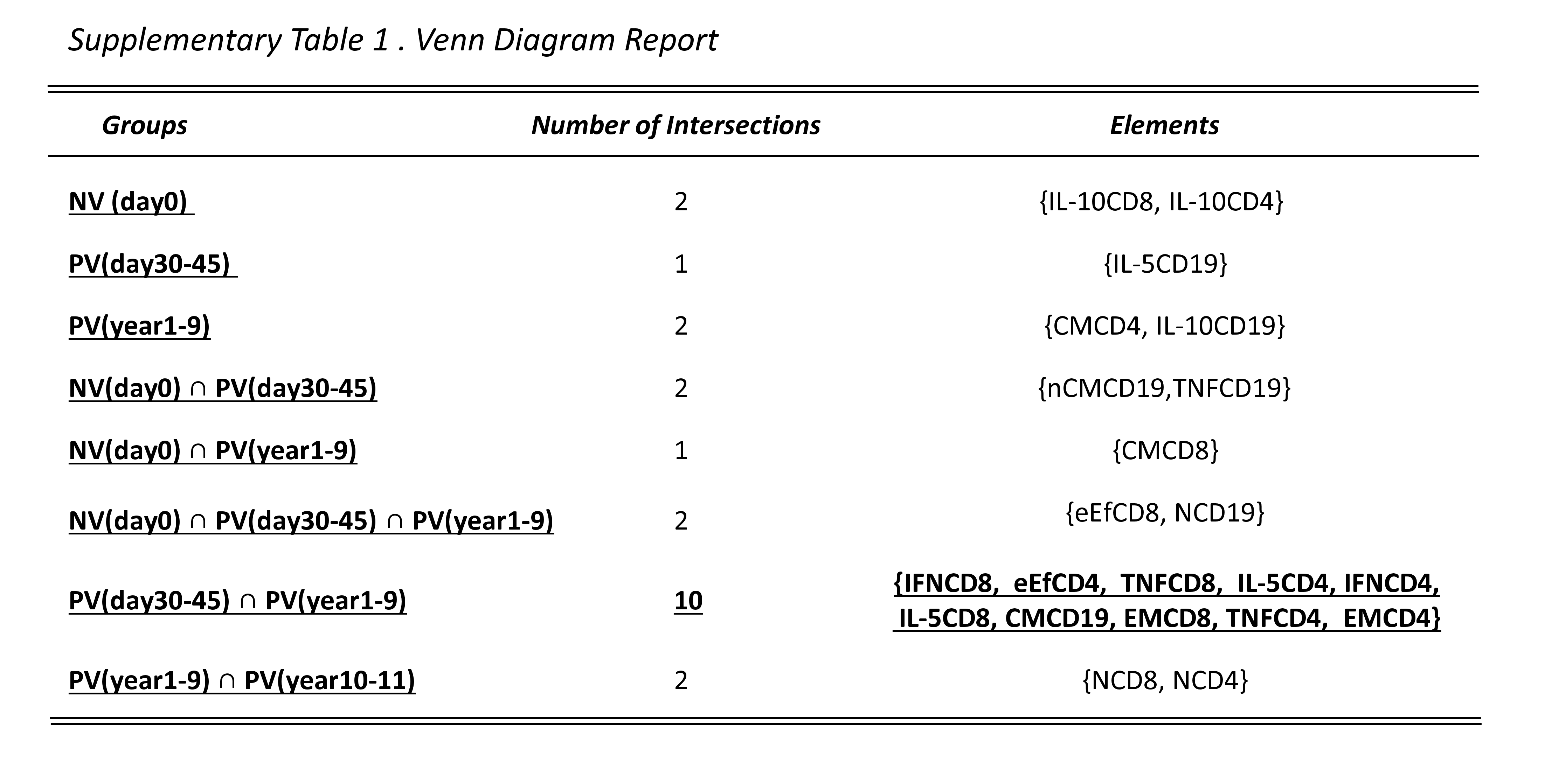

Supplement: S1 Table — Elements underscored and in bold correspond to biomarkers associated to protection. (TIFF) [file pntd.0006462.s001.tiff]
