# Supplementary material for: Multi-parameter approach to evaluate the timing of memory status after 17DD-YF primary vaccination
Source: PLoS Negl Trop Dis. 2018 Jun 7;12(6):e0006462. doi: 10.1371/journal.pntd.0006462 (PMC5991646; doi:10.1371/journal.pntd.0006462)
Supplement: S1 Checklist — (DOC) [file pntd.0006462.s002.doc]

STROBE Statement—Checklist of items that should be included in reports of ***cross-sectional studies***

|  | Item No | Recommendation |
| --- | --- | --- |
| **Title and abstract** | 1 | (a) Indicate the study’s design with a commonly used term in the title or the abstract **[First term of the title and first sentence of the abstract.]** |
|  |  | (*b*) Provide in the abstract an informative and balanced summary of what was done and what was found **[Second and last sentence of the abstract and also Author Summary.]** |
| Introduction | | |
| Background/rationale | 2 | Explain the scientific background and rationale for the investigation being reported **[Introduction, paragraphs 2, 3 and 4.]** |
| Objectives | 3 | State specific objectives, including any prespecified hypotheses **[Introduction, paragraphs 2, 3, 4 and 5.]** |
| Methods | | |
| Study design | 4 | Present key elements of study design early in the paper **[Materials and Methods, sections "Study population", "Plaque Reduction Neutralization Test (*PRNT*)" and "Long-term 17DD-YF Antigen Recall of Peripheral Blood Mononuclear Cells *in vitro*".]** |
| Setting | 5 | Describe the setting, locations, and relevant dates, including periods of recruitment, exposure, follow-up, and data collection **[Materials and Methods, section "Study population".]** |
| Participants | 6 | (*a*) Give the eligibility criteria, and the sources and methods of selection of participants **[Materials and Methods, section "Study population".]** |
| Variables | 7 | Clearly define all outcomes, exposures, predictors, potential confounders, and effect modifiers. Give diagnostic criteria, if applicable **[Materials and Methods, sections "Study population", "Plaque Reduction Neutralization Test (*PRNT*)","Long-term 17DD-YF Antigen Recall of Peripheral Blood Mononuclear Cells *in vitro*", “Analysis of 17DD-YF Memory-related phenotypic Biomarkers – T and B-cell surface molecules”, “Analysis of 17DD-YF Memory-related functional Biomarkers - Intracytoplasmic Cytokine pattern”, “Flow cytometric acquisition and analysis” and “Data Mining and analysis”.]** |
| Data sources/ measurement | 8* | For each variable of interest, give sources of data and details of methods of assessment (measurement). Describe comparability of assessment methods if there is more than one group **[Materials and Methods, sections "Study population", "Plaque Reduction Neutralization Test (*PRNT*)","Long-term 17DD-YF Antigen Recall of Peripheral Blood Mononuclear Cells *in vitro*", “Analysis of 17DD-YF Memory-related phenotypic Biomarkers – T and B-cell surface molecules”, “Analysis of 17DD-YF Memory-related functional Biomarkers - Intracytoplasmic Cytokine pattern”, “Flow cytometric acquisition and analysis” and “Data Mining and analysis”.]** |
| Bias | 9 | Describe any efforts to address potential sources of bias **[Materials and Methods, section "Study population".]** |
| Study size | 10 | Explain how the study size was arrived at **[Materials and Methods, section "Study population".]** |
| Quantitative variables | 11 | Explain how quantitative variables were handled in the analyses. If applicable, describe which groupings were chosen and why **[Materials and Methods, sections “Flow cytometric acquisition and analysis” and “Data Mining and analysis”.]** |
| Statistical methods | 12 | (*a*) Describe all statistical methods, including those used to control for confounding  **[Materials and Methods, sections “Flow cytometric acquisition and analysis” and “Data Mining and analysis”.]** |
|  |  | (*b*) Describe any methods used to examine subgroups and interactions **[Materials and Methods, sections “Flow cytometric acquisition and analysis” and “Data Mining and analysis”.]** |
|  |  | (*c*) Explain how missing data were addressed **[N/A]** |
|  |  | (*d*) If applicable, describe analytical methods taking account of sampling strategy **[N/A]** |
|  |  | (*e*) Describe any sensitivity analyses **[Materials and Methods, section “Data Mining and analysis”.]** |
| Results | | |
| Participants | 13* | (a) Report numbers of individuals at each stage of study—eg numbers potentially eligible, examined for eligibility, confirmed eligible, included in the study, completing follow-up, and analysed **[Materials and Methods, section "Study population".]** |
|  |  | (b) Give reasons for non-participation at each stage **[N/A]** |
|  |  | (c) Consider use of a flow diagram **[N/A]** |
| Descriptive data | 14* | (a) Give characteristics of study participants (eg demographic, clinical, social) and information on exposures and potential confounders **[Materials and Methods, section "Study population".]** |
|  |  | (b) Indicate number of participants with missing data for each variable of interest **[N/A]** |
| Outcome data | 15* | Report numbers of outcome events or summary measures **[Results, sections**  **“Overall 17DD-YF memory-related biomarker signatures at distinct time-points after primary vaccination”, “17DD-YF memory-related biomarker signatures according to the age at primary vaccination”, “Set of phenotypic/functional biomarkers useful to monitor the memory status following 17DD-YF primary vaccination”, “Major phenotypic/functional biomarkers useful to monitor the memory status following 17DD-YF primary vaccination” and “Changes in neutralizing antibody titers and phenotypic/functional memory-related biomarkers at distinct time-points after 17DD-YF primary vaccination”.]** |
| Main results | 16 | (*a*) Give unadjusted estimates and, if applicable, confounder-adjusted estimates and their precision (eg, 95% confidence interval) **[Results, sections“Overall 17DD-YF memory-related biomarker signatures at distinct time-points after primary vaccination”, “17DD-YF memory-related biomarker signatures according to the age at primary vaccination”, “Set of phenotypic/functional biomarkers useful to monitor the memory status following 17DD-YF primary vaccination”, “Major phenotypic/functional biomarkers useful to monitor the memory status following 17DD-YF primary vaccination” and “Changes in neutralizing antibody titers and phenotypic/functional memory-related biomarkers at distinct time-points after 17DD-YF primary vaccination”.]**  Make clear which confounders were adjusted for and why they were included **[N/A]** |
|  |  | (*b*) Report category boundaries when continuous variables were categorized **[Results, sections“Overall 17DD-YF memory-related biomarker signatures at distinct time-points after primary vaccination”, “17DD-YF memory-related biomarker signatures according to the age at primary vaccination”, “Set of phenotypic/functional biomarkers useful to monitor the memory status following 17DD-YF primary vaccination” and “Major phenotypic/functional biomarkers useful to monitor the memory status following 17DD-YF primary vaccination” .]** |
|  |  | (*c*) If relevant, consider translating estimates of relative risk into absolute risk for a meaningful time period **[N/A]** |
| Other  analyses | 17 | Report other analyses done—eg analyses of subgroups and interactions, and sensitivity analyses **[Results, sections “Set of phenotypic/functional biomarkers useful to monitor the memory status following 17DD-YF primary vaccination”, “Major phenotypic/functional biomarkers useful to monitor the memory status following 17DD-YF primary vaccination” and “Changes in neutralizing antibody titers and phenotypic/functional memory-related biomarkers at distinct time-points after 17DD-YF primary vaccination”.]** |
| Discussion | | |
| Key results | 18 | Summarise key results with reference to study objectives [**Discussion, paragraphs 2, 3, 5 and 6.]** |
| Limitations | 19 | Discuss limitations of the study, taking into account sources of potential bias or imprecision. Discuss both direction and magnitude of any potential bias **[Section "Study population".]** |
| Interpretation | 20 | Give a cautious overall interpretation of results considering objectives, limitations, multiplicity of analyses, results from similar studies, and other relevant evidence [**Discussion, paragraph 6.]** |
| Generalisability | 21 | Discuss the generalisability (external validity) of the study results **[Discussion, paragraphs 1, 2, 3, 4, 5 and 6.]** |
| Other information | | |
| Funding | 22 | Give the source of funding and the role of the funders for the present study and, if applicable, for the original study on which the present article is based **[within online submission system.]** |

*Give information separately for exposed and unexposed groups.

**Note:** An Explanation and Elaboration article discusses each checklist item and gives methodological background and published examples of transparent reporting. The STROBE checklist is best used in conjunction with this article (freely available on the Web sites of PLoS Medicine at http://www.plosmedicine.org/, Annals of Internal Medicine at http://www.annals.org/, and Epidemiology at http://www.epidem.com/). Information on the STROBE Initiative is available at www.strobe-statement.org.
